# Nicotine Increases Impulsivity and Decreases Willingness to Exert Cognitive Effort despite Improving Attention in “Slacker” Rats: Insights into Cholinergic Regulation of Cost/Benefit Decision Making

**DOI:** 10.1371/journal.pone.0111580

**Published:** 2014-10-29

**Authors:** Jay G. Hosking, Fred C. W. Lam, Catharine A. Winstanley

**Affiliations:** Department of Psychology, University of British Columbia, Vancouver, Canada; Universidade do Estado do Rio de Janeiro, Brazil

## Abstract

Successful decision making in our daily lives requires weighing an option’s costs against its associated benefits. The neuromodulator acetylcholine underlies both the etiology and treatment of a number of illnesses in which decision making is perturbed, including Alzheimer’s disease, attention-deficit/hyperactivity disorder, and schizophrenia. Nicotine acts on the cholinergic system and has been touted as a cognitive enhancer by both smokers and some researchers for its attention-boosting effects; however, it is unclear whether treatments that have a beneficial effect on attention would also have a beneficial effect on decision making. Here we utilize the rodent Cognitive Effort Task (rCET), wherein animals can choose to allocate greater visuospatial attention for a greater reward, to examine cholinergic contributions to both attentional performance and choice based on attentional demand. Following the establishment of baseline behavior, four drug challenges were administered: nicotine, mecamylamine, scopolamine, and oxotremorine (saline plus three doses for each). As per previous rCET studies, animals were divided by their baseline preferences, with “worker” rats choosing high-effort/high-reward options more than their “slacker” counterparts. Nicotine caused slackers to choose even fewer high-effort trials than at baseline, but had no effect on workers’ choice. Despite slackers’ decreased willingness to expend effort, nicotine improved their attentional performance on the task. Nicotine also increased measures of motor impulsivity in all animals. In contrast, scopolamine decreased animals’ choice of high-effort trials, especially for workers, while oxotremorine decreased motor impulsivity for all animals. In sum, the cholinergic system appears to contribute to decision making, and in part these contributions can be understood as a function of individual differences. While nicotine has been considered as a cognitive enhancer, these data suggest that its modest benefits to attention may be coupled with impulsiveness and decreased willingness to work hard, especially in individuals who are particularly sensitive to effort costs (i.e. slackers).

## Introduction

In our daily lives, we are often confronted with decisions that require weighing each option’s costs against its associated benefits. Disturbances in such cost/benefit decision making have been reported in populations of virtually every severe neuropsychiatric illness [1], [2], and can adversely affect the day-to-day lives of these individuals. Thus, laboratory models of decision making have been developed to characterize these deficits in humans and identify putative neurobiological mechanisms [3], [4], while animal models have allowed researchers to test the causative relationships between neural circuitry, neurochemistry, and choice [5]. These studies have yielded considerable converging data on contributions of cortico-limbic-striatal brain regions, as well as neuromodulatory influences, on decision making [6], [7].

Alterations in central cholinergic function underlie both the etiology and treatment of a number of illnesses in which decision making is perturbed, including Alzheimer’s disease, attention-deficit/hyperactivity disorder, and schizophrenia [8]–[12]. Interestingly, the most commonly reported cholinergic-driven improvements are within the attentional domain, a cognitive process long associated with central acetylcholine [13]. While recent studies have examined cholinergic contributions to decision making under risk and delay via multiple drugs [14], [15], and while one cholinergic agonist has been used to study effort-based decision making [16], [17], whether acetylcholine regulates decision making with attentional effort costs has yet to be investigated. As such, it is unclear whether treatments that have a beneficial effect on attention *per se* (e.g. nicotine, [18]) would also have a beneficial effect on choices related to those demand costs. Relatedly, cigarette smokers often claim that nicotine enhances their mental focus and performance, but such effects may be limited to specific cognitive domains or relevant only to a subsection of individuals [19].

Our group has recently validated a rodent Cognitive Effort Task (rCET), wherein animals can choose to allocate greater visuospatial attention for a greater reward, and this task provides measures of both attentional performance and choice based on attentional demand. Previous work with this task indicates that the neurochemical regulation of willingness to work can be dissociated from ability, and that baseline differences in the degree to which animals choose to apply cognitive effort to earn greater rewards is a key determinant of drug response. For example, the psychostimulant amphetamine caused hard-working animals to “slack off”, i.e. choose a greater proportion of trials with lower attentional demands, while “slacker” animals worked harder in response to the drug in the absence of any change in attentional accuracy [20]. The rCET is thus uniquely situated to dissociate acetylcholine’s influence on decision making under attentional costs from acetylcholine’s impact on attentional performance.

The goal of this study was therefore to examine how nicotinic and muscarinic acetylcholine receptor agonists and antagonists affected animals’ choice versus their attentional performance on the rCET, paying special consideration to these drugs’ interactions with animals’ existing choice preferences.

## Materials and Methods

### Subjects and ethics statement

Subjects were 24 male Long-Evans rats from Charles Rivers Laboratories (St. Constant, Quebec, Canada), each weighing 275–300 g at experimental commencement. Animals were food restricted to 14–16 g rat chow per day and thus maintained at ∼85% of their free-feeding weight. Water was available *ad libitum*. Animals were pair housed in a climate-controlled colony room on a 12 hr reverse light-dark cycle (lights off: 8∶00 am; temperature: 21°C). All housing and testing was in accordance with the Canadian Council of Animal Care, and all procedures were approved by the University of British Columbia’s Animal Care Committee.

### Behavioral testing

All testing took place within 12 standard five-hole operant chambers, each supplemented with two retractable response levers and enclosed in a ventilated, sound-attenuating cabinet (Med Associates Inc., Vermont, USA). The chambers were controlled by software written in Med-PC by CAW, running on an IBM-compatible computer.

### Habituation and pre-task training

All animals were habituated and trained for the rCET as previously described (see [20], including supplementary methods). In brief, and as per five-choice serial reaction time task (5CSRTT) training [21], animals first learned to make a nosepoke response in an illuminated aperture within 5 s to obtain a sucrose pellet reward (Bioserv, 45 mg). In subsequent sessions, animals were trained to respond on both of the response levers at a fixed ratio 1 (FR1) schedule for reward. Animals were then trained on a forced-choice variant of the rCET (55–60 sessions), wherein only a single lever extended, before the standard free-choice program.

### The rat Cognitive Effort Task (rCET)

**Figure 1:**
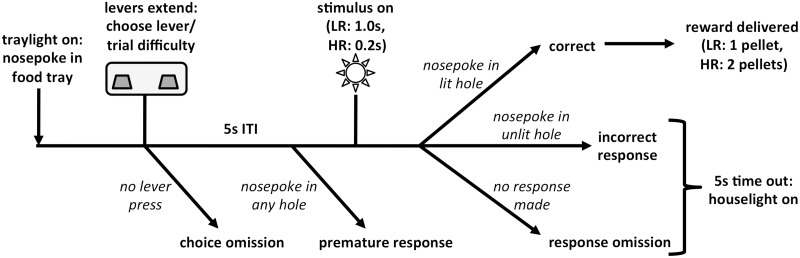
Schematic diagram showing the trial structure of the rCET. Trials began when the food-tray light illuminated. A nosepoke response in the food tray extinguished the light and extended the levers. Each lever was permanently designated to initiate either low-effort/low-reward (LR) or high-effort/high-reward (HR) trials. When animals pressed one of the levers, both levers retracted and a 5 s inter-trial interval (ITI) began. Following the ITI, one of the five stimulus lights briefly illuminated, 1.0 s for a LR trial and 0.2 s for a HR trial. If animals nosepoked in the previously illuminated aperture within 5 s (a correct response), they were rewarded 1 sugar pellet for a LR and 2 sugar pellets for a HR trial. A number of behaviors led to a 5 s time-out, signaled by house-light illumination: failure to make a lever response (choice omission); failure to withhold responding during the ITI (premature response); nosepoke in an unlit hole following the stimulus (incorrect response); failure to make a nosepoke response following the stimulus (response omission). Figure reprinted with permission from Cocker *et al* [20].

The rCET has been previously described in detail [20] and a schematic of the trial structure and subsequent reinforcement is presented in Figure 1. Briefly, animals were tested 4–5 days per week in 30 min sessions of no fixed trial limit. At the outset of training, the levers were permanently designated to initiate either low-effort/low-reward (LR) or high-effort/high-reward (HR) trials, and these designations were evenly counterbalanced across subjects.

New rCET trials were available when the food tray light was illuminated. A nosepoke in the food tray extinguished the light and extended the levers. Animals would then press one of the levers, thereby choosing a LR or HR trial, and this would cause both levers to retract and a 5 s inter-trial interval (ITI) to commence. After the ITI, one of the five stimulus lights briefly illuminated, with a stimulus duration of 1.0 s for LR trials and 0.2 s for HR trials. Animals were rewarded if they nosepoked the previously illuminated aperture within 5 s (a correct response), and received 1 sugar pellet for a LR trial and 2 sugar pellets for a HR trial. Upon reward delivery, the tray light again illuminated, thus signaling the opportunity to begin the next trial.

Trials went unrewarded for a number of reasons: if animals failed to make a lever response within 10 s (a choice omission); if animals nosepoked during the ITI (a premature response, a well-established measure of motor impulsivity [22]); if animals nosepoked in any aperture other than the one that was illuminated (an incorrect response); and if animals failed to nosepoke at the array within 5 s after stimulus-light illumination (a response omission). All such behaviors were punished with a 5 s time-out period, accompanied by illumination of the house light. During the time-out, new trials could not be initiated and thus reward could not be earned. Following the time-out, the house light extinguished and the tray light illuminated to signal that the rat could begin the next trial.

### Behavioral measurements for the rCET

Percent choice, rather than the absolute number of choices, was used to determine preference for lever/trial type, in order to minimize the influence of variation in the number of trials completed. Percent choice was calculated as follows: (number of choices of a particular lever/total number of choices) * 100. When baseline performance on the rCET was deemed statistically stable (i.e. no effect of session on repeated-measures ANOVA for choice, accuracy, and premature responding over the last three sessions; see “Data analysis” below), the mean choice of the HR option was 68%. Animals were grouped as “workers” if they chose HR for >70% of trials (n = 11) and as “slackers” if they chose HR for ≤70% of trials (n = 13). This subdivision was based on the mean split from the original rCET paper [20], where workers and slackers were categorized based on their preference for greater than or less than the average of 70% HR trials. To maintain consistency when discussing individual differences and to avoid arbitrary categorization, we therefore held the worker/slacker distinction at 70% HR trials for this study.

The following variables were also analyzed separately for LR and HR trials: percent accuracy ((number of correct responses/number of total responses made) * 100); percent premature responses ((number of premature responses/total number of trials initiated) * 100); latency to choose between the LR and HR levers (lever choice latency); latency to correctly nosepoke in the illuminated aperture (correct latency); latency to collect reward (collection latency); percent response omissions ((number of trials omitted/number of correct, incorrect, and omitted trials) * 100). Failures to choose a lever at the beginning of the trial (choice omissions) and the total number of completed trials were also analyzed.

### Pharmacological challenges

Drug doses were based on previous reports [14]. Upon stable baseline behavior, drugs were administered in the following order: the nicotinic acetylcholine receptor (nAChR) agonist nicotine (0, 0.1, 0.3, 1.0 mg/kg), the nAChR antagonist mecamylamine (0, 0.5, 1.0, 2.0 mg/kg), the muscarinic acetylcholine (mAChR) antagonist scopolamine (0, 0.03, 0.1, 0.3 mg/kg), and the mAChR agonist oxotremorine (0, 0.01, 0.03, 0.1 mg/kg). Nicotine and mecamylamine were purchased from Sigma-Aldrich Canada (Oakville, ON, Canada), whereas scopolamine and oxotremorine were purchased from Tocris (Minneapolis, MN, USA). All drugs were dissolved in 0.9% sterile saline and administered in a volume of 1 ml/kg via intraperitoneal injection.

All drugs were prepared fresh daily, and administration adhered to a digram-balanced Latin Square design (for doses A–D: ABCD, BDAC, CABD, DCBA, as per p.329 of [23]). The three-day injection schedule started with a baseline session, followed by a drug or saline injection session, and then by a non-testing day. Injections for nicotine and mecamylamine were administered 10 min before behavioral testing; scopolamine injections were administered immediately before testing; and oxotremorine injections were administered 15 min before testing. Animals were given a minimum of one week drug-free testing between compounds to minimize any carryover effects.

### Data analysis

All data were analyzed in SPSS (version 16.0; SPSS/IBM, Chicago, IL, USA). All variables expressed as a percentage were arcsine transformed to minimize artificial ceiling effects [24]. Baseline rCET data were analyzed using repeated-measures ANOVA with choice (two levels: LR or HR) and session (three levels: baseline sessions 1–3) as within-subjects factors. As discussed above, animals were categorized as workers and slackers at baseline, and group (two levels: worker or slacker) was therefore used as a between-subjects factor in all analyses. Groups proved extraordinarily stable across the experiment: at baseline and all saline conditions for drug challenges, workers chose a significantly greater percentage of HR trials than slackers (group: all Fs >19.809, p<0.001).

Pharmacological manipulations were again analyzed using repeated-measures ANOVA. For all drug challenges, dose (four levels: saline plus three drug doses) and choice were included as within-subjects factors, with group as a between-subjects factor. Any main effects of significance (p<0.05) were further analyzed via *post-hoc* one-way ANOVA or paired-samples t-tests. Any p-values>0.05 but <0.07 were reported as a statistical trend.

## Results

### Nicotine administration

#### Choice behavior, accuracy, and premature responses

**Figure 2:**
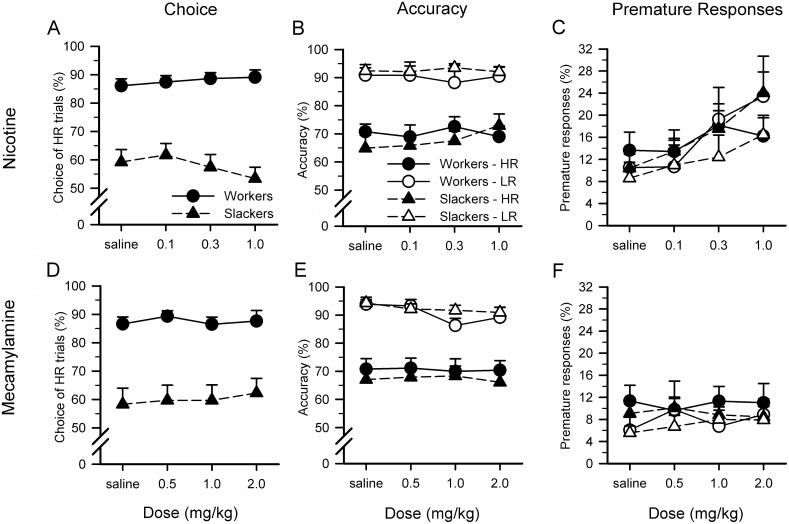
Nicotinic drug challenges during the rCET. (A) The nicotinic acetylcholine receptor (nAChR) agonist nicotine differentially affected choice of HR for workers and slackers, further decreasing choice of HR for slackers but having no effect on workers. (B) nicotine increased accuracy of HR for slackers, but had no effect on workers’ HR, or LR for all animals. (C) Nicotine increased premature responding for all animals across both trial types. (D) The nAChR antagonist mecamylamine did not affect animals’ choice on the rCET. (E) Mecamylamine caused a modest impairment to all animals’ accuracy on LR trials at the intermediate dose. (F) Mecamylamine had no effect on premature responding.

Baseline behavior has been discussed at length elsewhere [20], and as such will only be briefly addressed here. As demonstrated previously, animals chose high-effort/high-reward (HR) trials more than low-effort/low-reward (LR) trials (saline only–choice: F_1,22_ = 71.338, p<0.001), and workers continued to choose a significantly higher proportion of HR than slackers (saline only–group: F_1,22_ = 28.445, p<0.001). The nicotinic acetylcholine receptor (nAChR) agonist nicotine differentially affected choice of HR for workers and slackers (Figure 2a; dose: F_3,66_ = 0.377, NS; dose×group: F_3.66_ = 3.446, p = 0.022), further decreasing choice of HR for slackers but having no effect on workers (slackers only–dose: F_3,36_ = 4.300, p = 0.011; –saline vs 1.0 mg/kg–dose: F_1,12_ = 5.376, p = 0.039; –saline vs 0.1 mg/kg/−saline vs 0.3 mg/kg/workers only: all Fs<1.285, NS).

As expected, animals displayed higher accuracy on LR versus HR trials (saline only–choice: F_1,22_ = 62.446, p<0.001), indicating that HR trials were indeed more cognitively demanding. As seen in previous cohorts, workers and slackers performed the task equally well (saline only–choice×group/group: all Fs<0.499, NS), despite workers choosing HR proportionately more, and thus suggesting that differences in choice preference were not a direct result of differences in animals’ visuospatial attentional ability. Despite decreasing choice of HR for slackers, nicotine increased accuracy of HR for slackers, but had no effect on workers’ HR, or LR for all animals (Figure 2b; choice: F_1,22_ = 142.371, p<0.001; dose/dose×group/choice×dose/choice×dose×group: all Fs<0.841, NS; HR only–dose: F_3,66_ = 1.739, NS; –dose×group: F_3.66_ = 2.853, p = 0.044; –slackers only–dose: F_3,36_ = 3.208, p = 0.034; –saline vs 1.0 mg/kg: F_1,12_ = 8.388, p = 0.013; –saline vs 0.1 mg/kg/−saline vs 0.3 mg/kg: all Fs<1.007, NS; HR–workers only/LR: all Fs<1.064, NS).

As seen in previous cohorts, premature responding was higher for HR versus LR trials (saline only–choice: F_1,22_ = 7.384, p = 0.013). There were no differences in the level of premature responding between workers and slackers (saline only–choice×group/group: all Fs<0.377, NS), indicating that choice preference was not guided by motor impulsivity. Nicotine increased premature responding for all animals across both trial types (Figure 2c; dose: F_3,66_ = 5.287, p = 0.003; dose×group/choice×dose/choice×dose×group: all Fs<0.894, NS).

#### Other behavioral measures

Nicotine had no effect on the latency to choose a lever, nosepoke at the array or collect reward (dose/dose×group/choice×dose/choice×dose×group: all Fs<2.297, NS). As consistently seen with the rCET, both workers and slackers collected reward faster following a successful HR versus LR trial (choice: F_1,22_ = 4.393, p = 0.048; choice×group/group: all Fs<1.503, NS), suggesting that all animals anticipated a larger reward following the successful completion of HR, but slackers still chose proportionately fewer of these trials than workers. Nicotine dose-dependently increased choice omissions (dose: F_3,66_ = 14.429, p<0.001) but decreased response omissions (dose: F_3,66_ = 6.912, p<0.001) for all animals across both trial types (dose×group/choice×dose/choice×dose×group: all Fs<2.146, NS), and decreased the number of completed trials for all animals by ∼20% (dose: F_3,66_ = 20.042, p<0.001; dose×group: F_3,66_ = 0.380, NS).

### Mecamylamine administration

#### Choice behavior, accuracy, and premature responses

The nAChR antagonist mecamylamine did not affect animals’ choice on the rCET (Figure 2d; dose/dose×group: all Fs<1.562, NS). Mecamylamine caused a modest impairment to all animals’ accuracy on LR trials at the intermediate dose (Figure 2e; dose: F_3,66_ = 2.722, p = 0.051; dose×choice: F_3,66_ = 3.783, p = 0.014; LR only–dose: F_3,66_ = 3.896, p = 0.013; –saline vs 1.0 mg/kg–dose: F_1,22_ = 9.160, p = 0.006; –saline vs 0.5 mg/kg/−saline vs 2.0 mg/kg/HR only/dose×group/choice×dose×group: all Fs<3.514, NS). The drug had no effect on premature responding (Figure 2f; dose/dose×group/choice×dose/choice×dose×group: all Fs<0.646, NS).

#### Other behavioral measures

For all animals across both trial types, mecamylamine lengthened the latency to choose either the LR or HR lever (dose: F_3,66_ = 5.406, p = 0.009; dose×group/choice×dose/choice×dose×group: all Fs<1.637, NS) but did not affect correct or collection latencies (dose/dose×group/choice×dose/choice×dose×group: all Fs<1.043, NS). The drug did not affect response omissions (dose/dose×group/choice×dose/choice×dose×group: all Fs<0.939, NS) but increased the number of choice (lever) omissions (dose: F_3,66_ = 9.172, p<0.001; dose×group: F_3,66_ = 1.588, NS) and decreased the number of completed trials for all animals by ∼10% (dose: F_3,66_ = 8.716, p = 0.001; dose×group: F_3,66_ = 0.682, NS).

### Scopolamine administration

#### Choice behavior, accuracy, and premature responses

**Figure 3:**
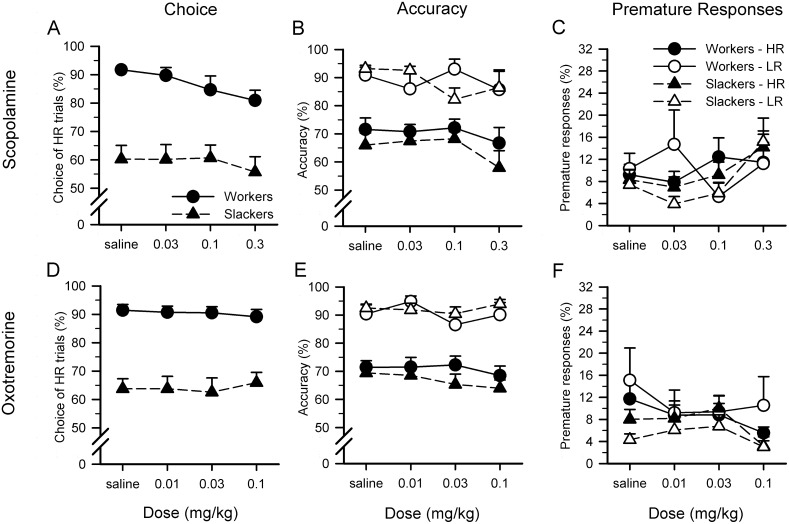
Muscarinic drug challenges during the rCET. (A) The muscarinic acetylcholine receptor (mAChR) antagonist scopolamine decreased all animals’ choice of HR. When examined separately, scopolamine decreased workers’ HR choice but had no effect on slackers’ choice. (B, C) The drug had no effect on animals’ accuracy or premature responding. (D, E) The mAChR agonist oxotremorine had no effect on animals’ choice or accuracy. (F) Oxotremorine did, however, decrease premature responding for all animals across both trial types.

The muscarinic acetylcholine receptor (mAChR) antagonist scopolamine decreased all animals’ choice of HR (Figure 3a; dose: F_3,66_ = 4.052, p = 0.011; dose×group: F_3,66_ = 1.393, NS; group: F_1,22_ = 27.043, p<0.001). When examined separately, scopolamine decreased workers’ HR choice (dose: F_3,30_ = 4.927, p = 0.007; saline vs 0.3 mg/kg–dose: F_1,10_ = 11.971, p = 0.006; saline vs 0.03 mg/kg/saline vs 0.1 mg/kg: all Fs<2.871, NS) but had no effect on slackers’ choice (dose: F_3,36_ = 0.526, NS). The drug had no effect on animals’ accuracy or premature responding (Figure 3b–c; dose/dose×group/choice×dose/choice×dose×group/LR only/HR only: all Fs<2.417, NS).

#### Other behavioral measures

Scopolamine had an inverted-U-shaped effect on the time taken to choose between LR and HR levers/options, with the lowest dose lengthening choice latency (dose: F_3,66_ = 4.843, p = 0.004; dose×group/choice×dose/choice×dose×group: all Fs<1.437, NS; saline vs 0.03 mg/kg–dose: F_1,22_ = 7.051, p = 0.014; 0.03 mg/kg vs 0.3 mg/kg–dose: F_1,22_ = 10.727, p = 0.003; 0.03 mg/kg vs 0.1 mg/kg–dose: F_1,22_ = 3.826, p = 0.063; saline vs 0.1 mg/kg/saline vs 0.3 mg/kg: all Fs<2.961, NS). Scopolamine also significantly lengthened all animals’ correct latency (dose: F_3,66_ = 7.255, p = 0.004; choice×dose: F_3,66_ = 3.097, p = 0.066; LR only–dose: F_3,66_ = 9.153, p = 0.002; HR only/dose×group/choice×dose×group: all Fs<1.469, NS) but had no effect on collection latency (dose/dose×group/choice×dose/choice×dose×group: all Fs<1.166, NS). The drug significantly increased both response omissions (dose: F_3,66_ = 47.154, p<0.001; dose×group: F_3,66_ = 2.805, p = 0.046; slackers only/workers only–dose: all Fs>13.879, p<0.001; choice×dose/choice×dose×group: all Fs<0.938, NS) and choice omissions (dose: F_3,66_ = 26.830, p<0.001; dose×group: F_3,66_ = 1.353, NS) and profoundly decreased all animals’ completed trials by over 65% (dose: F_3,66_ = 121.079, p<0.001; dose×group: F_3,66_ = 0.467, NS).

### Oxotremorine administration

#### Choice behavior, accuracy, and premature responses

The mAChR agonist oxotremorine had no effect on animals’ choice or accuracy (Figure 3d–e; dose/dose×group/choice×dose/choice×dose×group/LR only/HR only: all Fs<2.122, NS). Oxotremorine did, however, decrease premature responding for all animals across both trial types (Figure 3f; dose: F_3,66_ = 3.045, p = 0.035; saline vs 0.1 mg/kg–dose: F_1,22_ = 6.214, p = 0.021; saline vs 0.01 mg/kg/saline vs 0.03 mg/kg/dose×group/choice×dose/choice×dose×group: all Fs<1.052, NS).

#### Other behavioral measures

Oxotremorine lengthened choice latency for all animals across both trial types (dose: F_3,66_ = 7.408, p = 0.002; dose×group/choice×dose/choice×dose×group: all Fs<1.900, NS) but had no effect on correct or collection latencies (dose/dose×group/choice×dose/choice×dose×group: all Fs<1.925, NS). The drug increased both response omissions and choice omissions (dose: all Fs>9.026, p<0.001; dose×group/choice×dose/choice×dose×group: all Fs<2.002, NS), and also decreased completed trials by ∼40% (dose: F_3,66_ = 36.876, p<0.001; dose×group: F_3,66_ = 0.260, NS).

## Discussion

Here we show for the first time the influence of cholinergic functioning on decision making with attentional effort costs. The nAChR agonist nicotine decreased choice of high-effort/high-reward (HR) trials for “slacker” rats, despite a modest improvement in these animals’ performance (i.e. accuracy), whereas the drug did not affect workers’ choice. In contrast to its differential choice effects for workers versus slackers, nicotine increased motor impulsivity (i.e. premature responding) for all animals. Interestingly, the mAChR antagonist scopolamine also decreased choice of HR, particularly for workers, without any concomitant effects on performance or motor impulsivity. Finally, the mAChR agonist oxotremorine had no effect on choice but dose-dependently decreased impulsive responding. Taken together, these data support recent findings that nicotinic and muscarinic cholinergic systems subserve cost/benefit decision making [14], [15], and further demonstrate that acetylcholine’s influence on choice can be dissociated from its effects on attentional performance and motor impulsivity.

Central acetylcholine largely originates from the basal forebrain and pons, and projects to a diffuse set of targets in the central nervous system, including the prefrontal cortex, limbic regions, and the midbrain dopaminergic system [25]; a small population of cholinergic interneurons is also located in the striatum and projects locally, and thus acetylcholine exerts modulatory control over both dopamine’s midbrain source and its striatal targets [26], [27]. Broadly speaking, then, central cholinergic systems are excellently placed to both directly and indirectly contribute to the previously established “cortico-limbic-striatal” circuitry that underlies cost/benefit decision making [6]. Moreover, these distinct cholinergic pathways, for example to prefrontal cortex versus striatum, may make their own unique contributions to the decision-making process.

Pharmacological studies of cholinergic contributions to decision making have primarily used delay- and risk-discounting tasks, wherein the costs of the HR option were adjusted across blocks within each session [14]. On the risk-discounting task, nicotine increased choice of HR when costs ascended across blocks, whereas it decreased choice of HR when costs descended across blocks, indicating that the drug impaired animals’ behavioral flexibility. Scopolamine robustly decreased choice of HR on both tasks. Only null effects on decision making have been reported for mecamylamine and oxotremorine (for a review, see [28]), despite their nonspecific motor effects indicating a physiologically relevant dose range. These parallel the current data and suggest that these drugs may not be ideal for systemic manipulations of cost/benefit decision-making tasks, although they may be useful for injection into specific brain regions. As a comparison, the muscarinic agonist pilocarpine decreased choice of high-effort options on a well-established physical effort task when it was injected into the nucleus accumbens [16], but had less choice-specific effects when administered systemically [17]. This laboratory’s physical effort tasks were foundational to the study of effort-based decision making (e.g. [29]), and a pharmacological examination of those tasks would be of great relevance to the field. Furthermore, these results suggest dissociable contributions for striatal versus prefrontal cholinergic projections, a hypothesis that should be explored in the future.

In addition to its putative influence on decision making, acetylcholine’s role in attentional processes has also been well described (for a review, see [13]). For example, basal forebrain outputs to the sensory cortex increase the salience of objects by enhancing the reliability of sensory coding [30], while cholinergic contributions to the parietal and frontal lobes mediate shifting attention [31] and sustained attention [32], [33], respectively. Human studies of attention and acetylcholine generally correspond with this animal research [34], [35]. Taken together, acetylcholine appears intrinsically linked to the construct of attention and its various subcomponents, including salience, shift, and sustained effort.

As such, parsing acetylcholine’s contributions to both attention and decision making is essential to interpreting any manipulations of the cholinergic system. A substantial number of previous nicotine studies utilized the rodent Five-Choice Serial Reaction-Time Task (5CSRTT), the precursor to the rCET, which differs from the current task only in its lack of LR/HR options (thus having only a single stimulus duration and reward rate; [22]). In these 5CSRTT studies, systemic nicotine’s effect on animals’ accuracy was subtle, typically only benefitting performance under sub-optimal conditions such as when the basal forebrain was lesioned [18], when task difficulty was increased [36], or when using an inbred rat strain (versus the outbred strain of the current study; [37]). In addition to these minimal effects on accuracy, nicotine has also been reported to increase impulsive responding [36], [38], [39]. Taken together, these data imply that central cholinergic functioning already resides near an optimal level for attentional performance and inhibitory control.

In the current study, nicotine increased accuracy only for slackers on HR trials, and *prima facie* this may suggest that slackers suffer some performance impairment versus their worker counterparts. However, as discussed in detail elsewhere [20], [40], workers’ and slackers’ accuracy is equivalent at baseline, all animals demonstrate sensitivity to the task’s contingencies, and thus slackers’ choice of fewer HR trials is not simply dependent upon weaker performance or a failure to acquire the task. Furthermore, if nicotinic agonism was solely influencing attention on the task (and not decision making), then any benefits to HR performance should have been accompanied by increased choice of HR; instead, nicotine decreased HR choice while simultaneously increasing HR accuracy for slackers, suggesting that its effects on choice were separate from those on attention. One possibility is that striatal acetylcholine may be more heavily involved in the choice process, whereas prefrontal acetylcholine is predominantly involved in attentional performance on the task. Similarly, scopolamine decreased workers’ choice of HR but had no significant effect on accuracy. This lack of effect on accuracy stands in contrast to some 5CSRTT literature [41], [42], and may be the result of additional training for the rCET animals and differences in dosing methodology [43], [44]. Altogether, it appears that acetylcholine manipulations affect multiple subsystems, including those that underlie decision making, attention, and impulsivity.

Nicotine’s apparent lack of effect on workers’ choice is most readily interpreted by considering pharmacological results as a function of individual differences. Interactions between animals’ choice preferences and experimental manipulations have been previously reported for this task and cannot be explained by regression to the mean or indifference to the task’s choices [20], [40]. As discussed with amphetamine’s effects (see supplementary data of [20]), the current data suggest an inverted-U function of basal cholinergic tone versus choice of HR trials, upon which agonism would cause a rightward shift and antagonism a leftward shift; contrary to the monoamine systems, these data predict that slackers sit to the right of the apex on such a curve, hence a stronger choice effect for cholinergic agonism, while workers sit to the left of the curve, hence a stronger choice effect for cholinergic antagonism. A similar hypothesis was recently put forward by Mendez *et al.* [14], and directly testing such hypotheses of basal cholinergic and catecholamine functioning versus choice preference will require future *in vivo* behavioral recordings, such as via microdialysis or microelectrode array [45], [46]. As partial support of this, at least one study has demonstrated a relationship between DA-mediated activity in the nucleus accumbens and individual differences in willingness to exert physical effort [47].

In light of the current and previous data, some tentative, testable models of acetylcholine’s specific contribution to decision making can be made; these models are not mutually exclusive and may in fact complement one another. First, acetylcholine may indirectly influence choice via its interactions with the midbrain dopaminergic system [26], [27]. Some support for this theory can be observed in the general, but not absolute, congruency of effects for dopamine versus acetylcholine pharmacology on discounting tasks [28]: dopaminergic and cholinergic agonists tend to have the same effect on choice, and antagonists for each neuromodulator also tend to affect choice similarly. This is perhaps unsurprising, given the tightly linked nature of acetylcholine and dopamine in the striatum [25]. However, cholinergic contributions to decision making are not exclusively driven by dopaminergic interactions, as dopamine antagonists have no measurable effect on choice in the rCET (Hosking *et al.*, in press) and, as previously discussed, prefrontal versus striatal acetylcholine likely have dissociable contributions to behavior. Also, amphetamine (which potentiates dopaminergic functioning) has the opposite choice effects to nicotine, instead causing workers to “slack off” and slackers to “work harder” [20]. Second, acetylcholine may in part underlie animals’ ability to select and/or update their choice behavior; cholinergic agonism would thus render animals behaviorally inflexible, whereas antagonism would lead to behavioral indifference. This is supported both by previous results [14] and the current data: nicotine arguably exacerbated animals’ existing choice preferences and decreased sampling of animals’ less preferred option, whereas scopolamine drove all animals toward equivalent choice of LR versus HR and more greatly affected workers, whose preference was further from indifference. Third, acetylcholine may influence decision making via attentional processes, such as increasing the salience of the task’s objective and subjective properties. Such an interpretation could equally explain nicotine’s exacerbation of existing preferences on the rCET, when salience is increased, and scopolamine’s drive to indifference, when salience is decreased. Fourth, as cortical ACh efflux is known to track the amount of attentional effort exerted rather than attentional performance *per se* [32], [33], nicotine may have artificially inflated the sense of total effort expended in a rCET session, independent of its actual effects on attentional performance. This theory would suggest that animals more sensitive to the attentional effort exertion (i.e. slackers) would be more strongly affected by the drug, and indeed this is supported by the current data. Conversely, scopolamine could have increased the sense of effort expenditure to a greater degree in workers rather than slackers, thereby leading to the observed decrease in effortful choice predominantly in this harder-working group. Further disentangling these putative contributions of acetylcholine to decision making, for example by elucidating cortical versus striatal cholinergic influence on choice at baseline and in response to drug challenge, will be a focus of future research utilizing the rCET.

In sum, it appears that both nicotinic and muscarinic cholinergic systems contribute to cost/benefit decision making, and in part their contributions can be understood as a function of individual differences. While nicotine has been considered as a cognitive enhancer by both smokers and researchers [19], [48], [49], these data suggest that its modest benefits to attention may be coupled with impulsiveness and decreased willingness to work hard, especially in individuals who are particularly sensitive to effort costs (i.e. slackers). Nicotine may therefore produce a subjective feeling of increased output or task engagement, while actually producing a decrease in application. Novel therapeutic interventions may therefore be best understood by simultaneously studying multiple cognitive constructs such as decision making, attention, and impulsivity.

## Supporting Information

File S1 All relevant data in tables. **Table S1.** Nicotine. **Table S2.** Mecamylamine. **Table S3.** Scopolamine. **Table S4.** Oxotremorine.
(DOCX)
