# Supplementary material for: Nicotine Increases Impulsivity and Decreases Willingness to Exert Cognitive Effort despite Improving Attention in “Slacker” Rats: Insights into Cholinergic Regulation of Cost/Benefit Decision Making
Source: PLoS One. 2014 Oct 29;9(10):e111580. doi: 10.1371/journal.pone.0111580 (PMC4213040; doi:10.1371/journal.pone.0111580)
Supplement: File S1 — All relevant data in tables. Table S1. Nicotine. Table S2. Mecamylamine. Table S3. Scopolamine. Table S4. Oxotremorine. (DOCX) [file pone.0111580.s001.docx]

**File S1 – Supplementary Materials – All relevant data in tables**

**Nicotine increases impulsivity and decreases willingness to exert cognitive effort despite improving attention in “slacker” rats: insights into cholinergic regulation of cost/benefit decision making.**

Jay G. Hosking, Fred C.W. Lam, and Catharine A. Winstanley, Ph.D.

*Department of Psychology, University of British Columbia, Vancouver, Canada*

Please see original article for full description of behavioral measures.

**Table S1. Nicotine**

*1.1 HR Choice (%)*

| group |  | saline | 0.1mg/kg | 0.3mg/kg | 1.0mg/kg |
| --- | --- | --- | --- | --- | --- |
| all | average | 71.55457041 | 73.46632522 | 71.71681331 | 69.77003736 |
|  | st.e | 3.790760524 | 3.5897853 | 4.127802666 | 4.42481166 |
|  |  |  |  |  |  |
| workers | average | 86.11079452 | 87.38650708 | 88.6366479 | 89.07629138 |
|  | st.e | 2.410459998 | 2.27073607 | 2.070198617 | 2.61474113 |
|  |  |  |  |  |  |
| slackers | average | 59.23776539 | 61.68770979 | 57.40003018 | 53.43397627 |
|  | st.e | 4.378094201 | 4.09122136 | 4.472322838 | 3.992150658 |

*1.2 Accuracy (%)*

*LR*

| group |  | saline | 0.1mg/kg | 0.3mg/kg | 1.0mg/kg |
| --- | --- | --- | --- | --- | --- |
| all | average | 91.69615158 | 91.5028921 | 91.08692825 | 91.33435143 |
|  | st.e | 1.686864633 | 2.393424436 | 2.884482694 | 1.665500086 |
|  |  |  |  |  |  |
| workers | average | 90.84836059 | 90.7909147 | 88.21304689 | 90.49233223 |
|  | st.e | 2.640855007 | 4.765356458 | 6.111427843 | 3.112513483 |
|  |  |  |  |  |  |
| slackers | average | 92.4135132 | 92.10533451 | 93.51867402 | 92.04682921 |
|  | st.e | 2.248894273 | 2.047322524 | 1.424593972 | 1.700561892 |

*HR*

| group |  | saline | 0.1mg/kg | 0.3mg/kg | 1.0mg/kg |
| --- | --- | --- | --- | --- | --- |
| all | average | 67.58351739 | 67.2742162 | 69.81204941 | 71.14464374 |
|  | st.e | 2.820613156 | 2.733238138 | 2.487929777 | 2.815322283 |
|  |  |  |  |  |  |
| workers | average | 70.78172943 | 68.9438239 | 72.56473678 | 68.99946791 |
|  | st.e | 2.702633916 | 4.206550114 | 3.534040853 | 3.864994805 |
|  |  |  |  |  |  |
| slackers | average | 64.87733797 | 65.86147122 | 67.48285241 | 72.95979252 |
|  | st.e | 4.655056646 | 3.687035094 | 3.479841632 | 4.112615039 |

*1.3 premature responses*

*LR*

| group |  | saline | 0.1mg/kg | 0.3mg/kg | 1.0mg/kg |
| --- | --- | --- | --- | --- | --- |
| all | average | 9.472739618 | 10.80689317 | 15.54818444 | 19.48084162 |
|  | st.e | 2.039208839 | 2.902776338 | 3.401271308 | 3.720400879 |
|  |  |  |  |  |  |
| workers | average | 10.50990021 | 10.64418079 | 19.24838061 | 23.40116501 |
|  | st.e | 2.978543708 | 4.835373287 | 5.761115325 | 7.303183719 |
|  |  |  |  |  |  |
| slackers | average | 8.595142194 | 10.94457288 | 12.41724922 | 16.46520824 |
|  | st.e | 2.884182337 | 3.650415759 | 3.968547503 | 3.518762342 |

*HR*

| group |  | saline | 0.1mg/kg | 0.3mg/kg | 1.0mg/kg |
| --- | --- | --- | --- | --- | --- |
| all | average | 11.88213513 | 13.44329217 | 17.84147965 | 20.45805848 |
|  | st.e | 2.017776249 | 2.156267634 | 2.443716507 | 2.633217136 |
|  |  |  |  |  |  |
| workers | average | 13.63430571 | 13.42605924 | 18.17057428 | 16.23204053 |
|  | st.e | 3.305020828 | 3.926793755 | 3.880562237 | 3.304602387 |
|  |  |  |  |  |  |
| slackers | average | 10.39952925 | 13.45787387 | 17.56301497 | 24.03391983 |
|  | st.e | 2.510911842 | 2.362018499 | 3.240115988 | 3.8091928 |

*1.4 Lever/choice latency (s)*

*LR*

| group |  | saline | 0.1mg/kg | 0.3mg/kg | 1.0mg/kg |
| --- | --- | --- | --- | --- | --- |
| all | average | 3.382717925 | 3.110107461 | 3.394549413 | 3.52657028 |
|  | st.e | 0.241942908 | 0.275167126 | 0.246945706 | 0.24216267 |
|  |  |  |  |  |  |
| workers | average | 3.359682121 | 3.314892395 | 3.610788581 | 3.637137405 |
|  | st.e | 0.442987511 | 0.564732326 | 0.487944221 | 0.439516128 |
|  |  |  |  |  |  |
| slackers | average | 3.40220976 | 2.936827901 | 3.211577808 | 3.433013481 |
|  | st.e | 0.262081506 | 0.193858057 | 0.204480851 | 0.264009426 |

*HR*

| group |  | saline | 0.1mg/kg | 0.3mg/kg | 1.0mg/kg |
| --- | --- | --- | --- | --- | --- |
| all | average | 2.944229538 | 3.105991955 | 3.011257962 | 3.250082172 |
|  | st.e | 0.162925927 | 0.209923383 | 0.184181123 | 0.198715686 |
|  |  |  |  |  |  |
| workers | average | 2.815971061 | 3.205487922 | 3.079068087 | 3.348985617 |
|  | st.e | 0.18466262 | 0.313617002 | 0.277226262 | 0.254313647 |
|  |  |  |  |  |  |
| slackers | average | 3.052755942 | 3.02180306 | 2.953880163 | 3.166394642 |
|  | st.e | 0.26027185 | 0.29204503 | 0.255427741 | 0.304710759 |

*1.5 Correct latency (s)*

*LR*

| group |  | saline | 0.1mg/kg | 0.3mg/kg | 1.0mg/kg |
| --- | --- | --- | --- | --- | --- |
| all | average | 0.501200439 | 0.496457453 | 0.462887139 | 0.491599277 |
|  | st.e | 0.0251019 | 0.029092054 | 0.017020368 | 0.026723161 |
|  |  |  |  |  |  |
| workers | average | 0.45434947 | 0.461821535 | 0.444524711 | 0.493220425 |
|  | st.e | 0.024380867 | 0.050790815 | 0.025131516 | 0.045972091 |
|  |  |  |  |  |  |
| slackers | average | 0.540843566 | 0.525764768 | 0.478424579 | 0.490227537 |
|  | st.e | 0.039047603 | 0.031930821 | 0.023147788 | 0.032192058 |

*HR*

| group |  | saline | 0.1mg/kg | 0.3mg/kg | 1.0mg/kg |
| --- | --- | --- | --- | --- | --- |
| all | average | 0.454087181 | 0.450720982 | 0.42946576 | 0.39033659 |
|  | st.e | 0.036885507 | 0.027398834 | 0.029414472 | 0.014532449 |
|  |  |  |  |  |  |
| workers | average | 0.38038296 | 0.407414737 | 0.397403036 | 0.390570826 |
|  | st.e | 0.017510569 | 0.025771897 | 0.023378328 | 0.021165439 |
|  |  |  |  |  |  |
| slackers | average | 0.516452291 | 0.487364728 | 0.456595757 | 0.390138391 |
|  | st.e | 0.062376674 | 0.044088896 | 0.05042129 | 0.020770352 |

*1.6 Collection latency (s)*

*LR*

| group |  | saline | 0.1mg/kg | 0.3mg/kg | 1.0mg/kg |
| --- | --- | --- | --- | --- | --- |
| all | average | 1.721804886 | 1.747712845 | 1.887755731 | 1.866590419 |
|  | st.e | 0.128359751 | 0.215367492 | 0.227663231 | 0.296070594 |
|  |  |  |  |  |  |
| workers | average | 1.596718857 | 1.588696185 | 2.046485445 | 1.515278783 |
|  | st.e | 0.167017484 | 0.280731014 | 0.461334896 | 0.159452656 |
|  |  |  |  |  |  |
| slackers | average | 1.82764691 | 1.882265404 | 1.753445972 | 2.16385411 |
|  | st.e | 0.191296178 | 0.324441402 | 0.17334417 | 0.525527601 |

*HR*

| group |  | saline | 0.1mg/kg | 0.3mg/kg | 1.0mg/kg |
| --- | --- | --- | --- | --- | --- |
| all | average | 1.421268404 | 1.333224781 | 1.477171443 | 1.422836806 |
|  | st.e | 0.08751985 | 0.063955177 | 0.159487123 | 0.101002574 |
|  |  |  |  |  |  |
| workers | average | 1.502161641 | 1.422744618 | 1.623240877 | 1.577743909 |
|  | st.e | 0.165863148 | 0.078487681 | 0.335361423 | 0.196325739 |
|  |  |  |  |  |  |
| slackers | average | 1.352820281 | 1.257477227 | 1.35357423 | 1.291761565 |
|  | st.e | 0.082784697 | 0.095321638 | 0.089013781 | 0.076128847 |

*1.7 Response omissions (%)*

*LR*

| group |  | saline | 0.1mg/kg | 0.3mg/kg | 1.0mg/kg |
| --- | --- | --- | --- | --- | --- |
| all | average | 7.689114746 | 5.448739248 | 5.964972265 | 3.923309542 |
|  | st.e | 1.290532805 | 1.364856202 | 1.741750106 | 1.508294102 |
|  |  |  |  |  |  |
| workers | average | 8.899533583 | 5.557635855 | 6.758732668 | 0.871212121 |
|  | st.e | 2.311413374 | 2.321987756 | 3.159124696 | 0.581493886 |
|  |  |  |  |  |  |
| slackers | average | 6.664914191 | 5.356595966 | 5.293328847 | 6.271076788 |
|  | st.e | 1.391133969 | 1.669799062 | 1.901410116 | 2.475370942 |

*HR*

| group |  | saline | 0.1mg/kg | 0.3mg/kg | 1.0mg/kg |
| --- | --- | --- | --- | --- | --- |
| all | average | 7.716087596 | 7.087416385 | 4.631777973 | 4.964107705 |
|  | st.e | 0.925801749 | 1.30228864 | 0.96830453 | 1.152850595 |
|  |  |  |  |  |  |
| workers | average | 7.593494525 | 6.105314308 | 5.119248817 | 4.141635695 |
|  | st.e | 1.573224012 | 1.421429675 | 1.509606941 | 1.231993702 |
|  |  |  |  |  |  |
| slackers | average | 7.819820195 | 7.918425834 | 4.219302643 | 5.660045559 |
|  | st.e | 1.133875752 | 2.109698947 | 1.29609034 | 1.882693314 |

*1.8 Lever/choice omissions*

| group |  | saline | 0.1mg/kg | 0.3mg/kg | 1.0mg/kg |
| --- | --- | --- | --- | --- | --- |
| all | average | 3.25 | 4.375 | 4 | 10.58333333 |
|  | st.e | 0.493618699 | 0.816080461 | 0.974307632 | 1.781463194 |
|  |  |  |  |  |  |
| workers | average | 2.909090909 | 4.363636364 | 2.818181818 | 12.81818182 |
|  | st.e | 0.666804393 | 1.122276317 | 0.784140315 | 3.106644439 |
|  |  |  |  |  |  |
| slackers | average | 3.538461538 | 4.384615385 | 5 | 8.692307692 |
|  | st.e | 0.730431771 | 1.21179281 | 1.656378503 | 1.94272019 |

*1.9 Completed trials*

| group |  | saline | 0.1mg/kg | 0.3mg/kg | 1.0mg/kg |
| --- | --- | --- | --- | --- | --- |
| all | average | 123.9583333 | 122.3333333 | 118.3333333 | 99.66666667 |
|  | st.e | 4.881375121 | 4.544333415 | 5.010375708 | 4.976272202 |
|  |  |  |  |  |  |
| workers | average | 120.0909091 | 119.3636364 | 114.6363636 | 99.36363636 |
|  | st.e | 7.30685176 | 5.618593689 | 7.364870828 | 8.141110855 |
|  |  |  |  |  |  |
| slackers | average | 127.2307692 | 124.8461538 | 121.4615385 | 99.92307692 |
|  | st.e | 6.6872955 | 7.047318233 | 6.985897318 | 6.39356802 |

**Table S2. Mecamylamine**

*1.1 HR Choice (%)*

| group |  | saline | 0.5mg/kg | 1.0mg/kg | 2.0mg/kg |
| --- | --- | --- | --- | --- | --- |
| all | average | 71.28493902 | 73.28622035 | 72.00078545 | 73.91033237 |
|  | st.e | 4.326594247 | 4.275281622 | 4.164773432 | 4.122509708 |
|  |  |  |  |  |  |
| workers HR | average | 86.5872019 | 89.34801374 | 86.51206219 | 87.62785975 |
|  | st.e | 2.48319631 | 1.923623409 | 2.478189288 | 3.691586351 |
|  |  |  |  |  |  |
| slackers HR | average | 58.33687043 | 59.69547209 | 59.72201283 | 62.30319381 |
|  | st.e | 5.604254106 | 5.336999086 | 5.443611862 | 5.102110407 |

*1.2 Accuracy (%)*

*LR*

| group |  | saline | 0.5mg/kg | 1.0mg/kg | 2.0mg/kg |
| --- | --- | --- | --- | --- | --- |
| all LR | average | 94.09676675 | 92.66592659 | 89.21204643 | 90.11243244 |
|  | st.e | 1.300928737 | 1.417959239 | 1.590291116 | 1.899924664 |
|  |  |  |  |  |  |
| workers LR | average | 93.85052203 | 93.26834781 | 86.30036543 | 89.18442045 |
|  | st.e | 2.506814932 | 2.315510357 | 2.54520264 | 3.561459601 |
|  |  |  |  |  |  |
| slackers LR | average | 94.30512767 | 92.15618555 | 91.6757765 | 90.89767336 |
|  | st.e | 1.244806367 | 1.812901137 | 1.810350836 | 1.927921481 |

*HR*

| group |  | saline | 0.5mg/kg | 1.0mg/kg | 2.0mg/kg |
| --- | --- | --- | --- | --- | --- |
| all HR | average | 68.77452308 | 69.38835862 | 69.10568803 | 68.08293395 |
|  | st.e | 2.784492656 | 2.73468008 | 2.74754511 | 2.699877067 |
|  |  |  |  |  |  |
| workers HR | average | 70.78395887 | 71.14760649 | 70.00099073 | 70.41118507 |
|  | st.e | 3.716964765 | 3.592483905 | 4.443344443 | 3.393259085 |
|  |  |  |  |  |  |
| slackers HR | average | 67.07423126 | 67.89976426 | 68.3481242 | 66.11287531 |
|  | st.e | 4.143373155 | 4.118138003 | 3.561734149 | 4.119269746 |

*1.3 premature responses*

*LR*

| group |  | saline | 0.5mg/kg | 1.0mg/kg | 2.0mg/kg |
| --- | --- | --- | --- | --- | --- |
| all LR | average | 5.791282656 | 8.11721602 | 7.403499221 | 8.354676262 |
|  | st.e | 1.233284006 | 2.558411398 | 1.789182295 | 1.80050294 |
|  |  |  |  |  |  |
| workers LR | average | 6.061720143 | 9.786096257 | 6.735666359 | 8.898071625 |
|  | st.e | 2.332463364 | 5.146516705 | 2.932317702 | 2.816162029 |
|  |  |  |  |  |  |
| slackers LR | average | 5.562450937 | 6.705086589 | 7.968588565 | 7.894880186 |
|  | st.e | 1.239464706 | 2.030234393 | 2.281695313 | 2.416686931 |

*HR*

| group |  | saline | 0.5mg/kg | 1.0mg/kg | 2.0mg/kg |
| --- | --- | --- | --- | --- | --- |
| all HR | average | 10.1154065 | 9.870560345 | 9.958022397 | 9.622722172 |
|  | st.e | 1.716656531 | 1.415774963 | 1.642170467 | 1.993590152 |
|  |  |  |  |  |  |
| workers HR | average | 11.33517924 | 9.613828338 | 11.30107526 | 11.01816791 |
|  | st.e | 2.848140761 | 2.159308474 | 2.673460416 | 3.486316924 |
|  |  |  |  |  |  |
| slackers HR | average | 9.083291097 | 10.08779512 | 8.82159305 | 8.441960396 |
|  | st.e | 2.124596156 | 1.948393817 | 2.065820387 | 2.288965411 |

*1.4 Lever/choice latency (s)*

*LR*

| group |  | saline | 0.5mg/kg | 1.0mg/kg | 2.0mg/kg |
| --- | --- | --- | --- | --- | --- |
| all LR | average | 3.304792801 | 3.223074334 | 3.553737108 | 3.637865416 |
|  | st.e | 0.185088981 | 0.219076213 | 0.208753874 | 0.249758736 |
|  |  |  |  |  |  |
| workers LR | average | 3.324914001 | 3.097472286 | 3.558317021 | 3.751635904 |
|  | st.e | 0.340599691 | 0.415420196 | 0.400456076 | 0.470835178 |
|  |  |  |  |  |  |
| slackers LR | average | 3.28776717 | 3.329352989 | 3.549861796 | 3.54159808 |
|  | st.e | 0.198411314 | 0.214676212 | 0.202778327 | 0.25045026 |

*HR*

| group |  | saline | 0.5mg/kg | 1.0mg/kg | 2.0mg/kg |
| --- | --- | --- | --- | --- | --- |
| all HR | average | 3.139204448 | 3.262695457 | 3.346498081 | 3.562707983 |
|  | st.e | 0.162385447 | 0.163442062 | 0.193279358 | 0.175384435 |
|  |  |  |  |  |  |
| workers HR | average | 3.232031186 | 3.332656407 | 3.228738486 | 3.540874082 |
|  | st.e | 0.234670786 | 0.226377841 | 0.254794811 | 0.259588995 |
|  |  |  |  |  |  |
| slackers HR | average | 3.060658746 | 3.20349773 | 3.446140815 | 3.581182823 |
|  | st.e | 0.230981428 | 0.240314794 | 0.290866937 | 0.247515401 |

*1.5 Correct latency (s)*

*LR*

| group |  | saline | 0.5mg/kg | 1.0mg/kg | 2.0mg/kg |
| --- | --- | --- | --- | --- | --- |
| all LR | average | 0.528543959 | 0.51586305 | 0.524314335 | 0.517618962 |
|  | st.e | 0.017755249 | 0.021985372 | 0.031204945 | 0.030389275 |
|  |  |  |  |  |  |
| workers LR | average | 0.500594916 | 0.45925124 | 0.49983904 | 0.458294195 |
|  | st.e | 0.022542562 | 0.026266097 | 0.049923537 | 0.020490722 |
|  |  |  |  |  |  |
| slackers LR | average | 0.55219315 | 0.563765352 | 0.5450242 | 0.567816842 |
|  | st.e | 0.025602442 | 0.028401753 | 0.040126768 | 0.050135669 |

*HR*

| group |  | saline | 0.5mg/kg | 1.0mg/kg | 2.0mg/kg |
| --- | --- | --- | --- | --- | --- |
| all HR | average | 0.476312545 | 0.460490003 | 0.458831263 | 0.486877392 |
|  | st.e | 0.025922567 | 0.01980483 | 0.018347501 | 0.020165376 |
|  |  |  |  |  |  |
| workers HR | average | 0.429842166 | 0.437431269 | 0.420116072 | 0.457409875 |
|  | st.e | 0.022836178 | 0.028964011 | 0.017019463 | 0.026050272 |
|  |  |  |  |  |  |
| slackers HR | average | 0.515633636 | 0.48000124 | 0.49159027 | 0.511811444 |
|  | st.e | 0.041580496 | 0.026965485 | 0.02813872 | 0.029109089 |

*1.6 Collection latency (s)*

*LR*

| group |  | saline | 0.5mg/kg | 1.0mg/kg | 2.0mg/kg |
| --- | --- | --- | --- | --- | --- |
| all LR | average | 1.720773069 | 1.515556243 | 1.586899277 | 1.577673247 |
|  | st.e | 0.126950111 | 0.076169312 | 0.145900334 | 0.139624817 |
|  |  |  |  |  |  |
| workers LR | average | 1.673858149 | 1.449775915 | 1.655596468 | 1.621694235 |
|  | st.e | 0.204646528 | 0.099614247 | 0.300935085 | 0.283761561 |
|  |  |  |  |  |  |
| slackers LR | average | 1.760470309 | 1.57121652 | 1.528770885 | 1.540424719 |
|  | st.e | 0.164953276 | 0.113881729 | 0.104167114 | 0.109329765 |

*HR*

| group |  | saline | 0.5mg/kg | 1.0mg/kg | 2.0mg/kg |
| --- | --- | --- | --- | --- | --- |
| all HR | average | 1.35655553 | 1.382634896 | 1.345544816 | 1.362312404 |
|  | st.e | 0.066824065 | 0.064648734 | 0.076493908 | 0.07458347 |
|  |  |  |  |  |  |
| workers HR | average | 1.299382157 | 1.445283924 | 1.398389173 | 1.4225539 |
|  | st.e | 0.074670444 | 0.095211319 | 0.134631698 | 0.121125651 |
|  |  |  |  |  |  |
| slackers HR | average | 1.404932999 | 1.329624181 | 1.300830359 | 1.31133883 |
|  | st.e | 0.106975483 | 0.088802001 | 0.086949418 | 0.094157981 |

*1.7 Response omissions (%)*

*LR*

| group |  | saline | 0.5mg/kg | 1.0mg/kg | 2.0mg/kg |
| --- | --- | --- | --- | --- | --- |
| all LR | average | 4.343223842 | 5.163690406 | 5.732845701 | 3.956180959 |
|  | st.e | 0.902841109 | 1.535204206 | 1.367571305 | 1.219812688 |
|  |  |  |  |  |  |
| workers LR | average | 3.02436126 | 2.651515152 | 3.024693666 | 1.542699725 |
|  | st.e | 1.208076828 | 1.424967578 | 1.193318171 | 1.246385237 |
|  |  |  |  |  |  |
| slackers LR | average | 5.459184488 | 7.289377159 | 8.024358962 | 5.998357388 |
|  | st.e | 1.276224283 | 2.46704088 | 2.158464857 | 1.84698654 |

*HR*

| group |  | saline | 0.5mg/kg | 1.0mg/kg | 2.0mg/kg |
| --- | --- | --- | --- | --- | --- |
| all HR | average | 5.873528458 | 6.203028466 | 5.623545269 | 4.944875784 |
|  | st.e | 1.029316771 | 1.105917004 | 0.932585127 | 0.662351341 |
|  |  |  |  |  |  |
| workers HR | average | 5.005224447 | 4.481012874 | 5.507318534 | 4.277762813 |
|  | st.e | 1.356390815 | 0.974237687 | 1.404094632 | 0.792505572 |
|  |  |  |  |  |  |
| slackers HR | average | 6.608247236 | 7.660118583 | 5.721890969 | 5.50935599 |
|  | st.e | 1.534123733 | 1.80898837 | 1.298286272 | 1.02545376 |

*1.8 Lever/choice omissions*

| group |  | saline | 0.5mg/kg | 1.0mg/kg | 2.0mg/kg |
| --- | --- | --- | --- | --- | --- |
| all | average | 2.708333333 | 3.083333333 | 4.125 | 7.791666667 |
|  | st.e | 0.588165801 | 0.789231571 | 0.730575761 | 1.227668886 |
|  |  |  |  |  |  |
| workers | average | 3.090909091 | 3.363636364 | 3.454545455 | 6 |
|  | st.e | 1.107450351 | 1.539024055 | 1.162783779 | 1.853743142 |
|  |  |  |  |  |  |
| slackers | average | 2.384615385 | 2.846153846 | 4.692307692 | 9.307692308 |
|  | st.e | 0.583298111 | 0.723649528 | 0.936336392 | 1.578641965 |

*1.9 Completed trials*

| group |  | saline | 0.5mg/kg | 1.0mg/kg | 2.0mg/kg |
| --- | --- | --- | --- | --- | --- |
| all | average | 129.375 | 129.0833333 | 121.5 | 117.4166667 |
|  | st.e | 3.453528179 | 3.484394125 | 3.816915061 | 4.479387844 |
|  |  |  |  |  |  |
| workers | average | 126.5454545 | 127.0909091 | 120.2727273 | 118.6363636 |
|  | st.e | 5.57325372 | 4.933106242 | 5.820170115 | 6.804106684 |
|  |  |  |  |  |  |
| slackers | average | 131.7692308 | 130.7692308 | 122.5384615 | 116.3846154 |
|  | st.e | 4.388437381 | 5.028322152 | 5.241128505 | 6.17743875 |

**Table S3. Scopolamine**

*1.1 HR Choice (%)*

| group |  | saline | 0.03mg/kg | 0.1mg/kg | 0.3mg/kg |
| --- | --- | --- | --- | --- | --- |
| all | average | 74.66783721 | 73.7269453 | 71.6872302 | 67.28122166 |
|  | st.e | 4.186654558 | 4.310731093 | 4.093839982 | 4.208832067 |
|  |  |  |  |  |  |
| workers HR | average | 91.72730254 | 89.74152121 | 84.67430605 | 80.94289143 |
|  | st.e | 0.989298472 | 2.789950179 | 4.873550837 | 3.584401443 |
|  |  |  |  |  |  |
| slackers HR | average | 60.23290501 | 60.1761503 | 60.69816603 | 55.72134723 |
|  | st.e | 4.842246309 | 5.184431916 | 4.538590973 | 5.415286051 |

*1.2 Accuracy (%)*

*LR*

| group |  | saline | 0.03mg/kg | 0.1mg/kg | 0.3mg/kg |
| --- | --- | --- | --- | --- | --- |
| all LR | average | 92.13986025 | 89.55864471 | 87.17094514 | 86.13425926 |
|  | st.e | 1.42063873 | 2.770416906 | 2.923084262 | 4.431117949 |
|  |  |  |  |  |  |
| workers LR | average | 90.88678998 | 85.995671 | 93.01948052 | 85.75757576 |
|  | st.e | 2.818309547 | 5.759634108 | 3.550707302 | 6.499275936 |
|  |  |  |  |  |  |
| slackers LR | average | 93.20015048 | 92.57346863 | 82.22218444 | 86.45299145 |
|  | st.e | 1.155954364 | 1.454087412 | 4.109917037 | 6.298690824 |

*HR*

| group |  | saline | 0.03mg/kg | 0.1mg/kg | 0.3mg/kg |
| --- | --- | --- | --- | --- | --- |
| all HR | average | 68.56693054 | 68.97234132 | 70.00367962 | 61.98588649 |
|  | st.e | 2.979621108 | 2.207314868 | 1.990637502 | 4.153534614 |
|  |  |  |  |  |  |
| workers HR | average | 71.58840522 | 70.74886605 | 72.11248125 | 66.734425 |
|  | st.e | 4.058830645 | 2.573345526 | 3.136694833 | 5.533495254 |
|  |  |  |  |  |  |
| slackers HR | average | 66.01029811 | 67.46912809 | 68.21930901 | 57.96789237 |
|  | st.e | 4.31395133 | 3.48711199 | 2.550506224 | 6.04309206 |

*1.3 premature responses*

*LR*

| group |  | saline | 0.03mg/kg | 0.1mg/kg | 0.3mg/kg |
| --- | --- | --- | --- | --- | --- |
| all LR | average | 8.746303667 | 8.871715637 | 5.584463052 | 13.38155241 |
|  | st.e | 1.945552005 | 3.077280301 | 1.516218968 | 3.314871751 |
|  |  |  |  |  |  |
| workers LR | average | 10.32328115 | 14.71861472 | 5.264245324 | 11.22294372 |
|  | st.e | 2.784162196 | 6.219235778 | 2.570073109 | 5.325135042 |
|  |  |  |  |  |  |
| slackers LR | average | 7.411938108 | 3.924339492 | 5.855416513 | 15.20806745 |
|  | st.e | 2.757555387 | 1.335720581 | 1.861108614 | 4.275511925 |

*HR*

| group |  | saline | 0.03mg/kg | 0.1mg/kg | 0.3mg/kg |
| --- | --- | --- | --- | --- | --- |
| all HR | average | 8.728148022 | 7.363062558 | 10.6854294 | 12.939327 |
|  | st.e | 1.258321685 | 1.327284339 | 1.999887689 | 2.27341268 |
|  |  |  |  |  |  |
| workers HR | average | 9.207817694 | 7.860552777 | 12.42688876 | 11.45969598 |
|  | st.e | 1.885165255 | 1.955796671 | 3.459039665 | 3.603423824 |
|  |  |  |  |  |  |
| slackers HR | average | 8.322273684 | 6.942109296 | 9.211886854 | 14.19132248 |
|  | st.e | 1.751238413 | 1.871955409 | 2.306651728 | 2.974449882 |

*1.4 Lever/choice latency (s)*

*LR*

| group |  | saline | 0.03mg/kg | 0.1mg/kg | 0.3mg/kg |
| --- | --- | --- | --- | --- | --- |
| all LR | average | 3.497535334 | 3.927387173 | 3.633409273 | 3.43642165 |
|  | st.e | 0.224978295 | 0.226324601 | 0.228144674 | 0.267973182 |
|  |  |  |  |  |  |
| workers LR | average | 3.811167658 | 3.896056097 | 3.963682217 | 3.363493998 |
|  | st.e | 0.391140842 | 0.45768724 | 0.400229352 | 0.285595674 |
|  |  |  |  |  |  |
| slackers LR | average | 3.232154136 | 3.953898084 | 3.353947552 | 3.498129663 |
|  | st.e | 0.241695539 | 0.182793977 | 0.238803637 | 0.44259412 |

*HR*

| group |  | saline | 0.03mg/kg | 0.1mg/kg | 0.3mg/kg |
| --- | --- | --- | --- | --- | --- |
| all HR | average | 3.143171469 | 3.518622599 | 2.979960542 | 2.5806962 |
|  | st.e | 0.167847197 | 0.192945865 | 0.19137165 | 0.148881216 |
|  |  |  |  |  |  |
| workers HR | average | 3.24030373 | 3.49453509 | 3.247848901 | 2.551955538 |
|  | st.e | 0.272478607 | 0.270167877 | 0.229168129 | 0.243439525 |
|  |  |  |  |  |  |
| slackers HR | average | 3.060982632 | 3.539004336 | 2.753285777 | 2.605015221 |
|  | st.e | 0.214727296 | 0.283172926 | 0.288470855 | 0.191145959 |

*1.5 Correct latency (s)*

*LR*

| group |  | saline | 0.03mg/kg | 0.1mg/kg | 0.3mg/kg |
| --- | --- | --- | --- | --- | --- |
| all LR | average | 0.513117268 | 0.543086299 | 0.594512797 | 0.787368604 |
|  | st.e | 0.025701454 | 0.031428378 | 0.029611118 | 0.080133841 |
|  |  |  |  |  |  |
| workers LR | average | 0.47738961 | 0.496222407 | 0.557437229 | 0.746242424 |
|  | st.e | 0.042602124 | 0.041809828 | 0.047033339 | 0.111939775 |
|  |  |  |  |  |  |
| slackers LR | average | 0.543348362 | 0.582740362 | 0.625884431 | 0.822167679 |
|  | st.e | 0.029857155 | 0.044458788 | 0.036929764 | 0.116902506 |

*HR*

| group |  | saline | 0.03mg/kg | 0.1mg/kg | 0.3mg/kg |
| --- | --- | --- | --- | --- | --- |
| all HR | average | 0.45655683 | 0.506325335 | 0.503392552 | 0.564775068 |
|  | st.e | 0.02265197 | 0.041196687 | 0.036791687 | 0.06144111 |
|  |  |  |  |  |  |
| workers HR | average | 0.423819619 | 0.459673587 | 0.448442796 | 0.53474972 |
|  | st.e | 0.027221457 | 0.051385068 | 0.025679068 | 0.076474887 |
|  |  |  |  |  |  |
| slackers HR | average | 0.484257547 | 0.54579989 | 0.549888499 | 0.590181132 |
|  | st.e | 0.03396478 | 0.062130551 | 0.062703443 | 0.095480322 |

*1.6 Collection latency (s)*

*LR*

| group |  | saline | 0.03mg/kg | 0.1mg/kg | 0.3mg/kg |
| --- | --- | --- | --- | --- | --- |
| all LR | average | 1.632363329 | 2.144284714 | 3.763092327 | 2.160352927 |
|  | st.e | 0.138097278 | 0.437891896 | 1.830827485 | 0.275131539 |
|  |  |  |  |  |  |
| workers LR | average | 1.705119408 | 2.7654242 | 5.980633117 | 2.412757576 |
|  | st.e | 0.28173589 | 0.937339961 | 3.979638631 | 0.518352858 |
|  |  |  |  |  |  |
| slackers LR | average | 1.570800492 | 1.61870515 | 1.886711659 | 1.946779763 |
|  | st.e | 0.104032591 | 0.09350514 | 0.230308401 | 0.264760594 |

*HR*

| group |  | saline | 0.03mg/kg | 0.1mg/kg | 0.3mg/kg |
| --- | --- | --- | --- | --- | --- |
| all HR | average | 1.376785967 | 1.509996445 | 1.393340918 | 1.614577767 |
|  | st.e | 0.064099097 | 0.061610137 | 0.081467104 | 0.142088419 |
|  |  |  |  |  |  |
| workers HR | average | 1.448471176 | 1.522249029 | 1.34170527 | 1.726541964 |
|  | st.e | 0.091180889 | 0.114427211 | 0.084134223 | 0.206213058 |
|  |  |  |  |  |  |
| slackers HR | average | 1.316129251 | 1.499628874 | 1.43703262 | 1.519838832 |
|  | st.e | 0.089482404 | 0.064646144 | 0.134707069 | 0.199553924 |

*1.7 Response omissions (%)*

*LR*

| group |  | saline | 0.03mg/kg | 0.1mg/kg | 0.3mg/kg |
| --- | --- | --- | --- | --- | --- |
| all LR | average | 5.526784449 | 9.24398456 | 18.33220634 | 38.71688191 |
|  | st.e | 1.088128516 | 1.924480883 | 3.054943037 | 3.529581656 |
|  |  |  |  |  |  |
| workers LR | average | 5.569324466 | 6.43037518 | 19.14491808 | 44.82290437 |
|  | st.e | 1.705985812 | 2.165865822 | 4.966581748 | 4.692854541 |
|  |  |  |  |  |  |
| slackers LR | average | 5.49078905 | 11.62473096 | 17.64452717 | 33.55024752 |
|  | st.e | 1.461913217 | 2.960889211 | 3.942586092 | 4.86821671 |

*HR*

| group |  | saline | 0.03mg/kg | 0.1mg/kg | 0.3mg/kg |
| --- | --- | --- | --- | --- | --- |
| all HR | average | 6.156679614 | 9.034606584 | 18.77263632 | 34.83308371 |
|  | st.e | 0.936831165 | 1.410183819 | 2.863144023 | 3.732949218 |
|  |  |  |  |  |  |
| workers HR | average | 4.854614687 | 7.022883704 | 12.31720329 | 37.8259813 |
|  | st.e | 1.225635748 | 1.480452348 | 2.507932766 | 5.666793004 |
|  |  |  |  |  |  |
| slackers HR | average | 7.25842686 | 10.73683364 | 24.23492581 | 32.30063191 |
|  | st.e | 1.351151003 | 2.228084485 | 4.375724936 | 5.049616428 |

*1.8 Lever/choice omissions*

| group |  | saline | 0.03mg/kg | 0.1mg/kg | 0.3mg/kg |
| --- | --- | --- | --- | --- | --- |
| all | average | 2.75 | 11.41666667 | 16.83333333 | 16.16666667 |
|  | st.e | 0.400407401 | 1.698162548 | 1.895519762 | 1.42908031 |
|  |  |  |  |  |  |
| workers | average | 2.181818182 | 7.818181818 | 16.63636364 | 13.90909091 |
|  | st.e | 0.629836657 | 1.872374194 | 2.090118428 | 2.364335561 |
|  |  |  |  |  |  |
| slackers | average | 3.230769231 | 14.46153846 | 17 | 18.07692308 |
|  | st.e | 0.495542259 | 2.458932916 | 3.102934922 | 1.619043475 |

*1.9 Completed trials*

| group |  | saline | 0.03mg/kg | 0.1mg/kg | 0.3mg/kg |
| --- | --- | --- | --- | --- | --- |
| all | average | 127.4166667 | 99.95833333 | 62.66666667 | 41.91666667 |
|  | st.e | 3.942483523 | 4.923501452 | 5.245310765 | 2.737234314 |
|  |  |  |  |  |  |
| workers | average | 127.4545455 | 104.1818182 | 60.72727273 | 42.81818182 |
|  | st.e | 4.22624621 | 6.794139429 | 5.991040142 | 3.893626918 |
|  |  |  |  |  |  |
| slackers | average | 127.3846154 | 96.38461538 | 64.30769231 | 41.15384615 |
|  | st.e | 6.511861427 | 7.134727693 | 8.459090555 | 3.96433807 |

**Table S4. Oxotremorine**

*1.1 HR Choice (%)*

| group |  | saline | 0.01mg/kg | 0.03mg/kg | 0.1mg/kg |
| --- | --- | --- | --- | --- | --- |
| all | average | 76.46702724 | 76.10918683 | 75.39240542 | 76.56195973 |
|  | st.e | 3.545702914 | 3.770034593 | 4.053850931 | 3.297052159 |
|  |  |  |  |  |  |
| workers HR | average | 91.44808964 | 90.70388173 | 90.53486255 | 89.13804843 |
|  | st.e | 2.008917051 | 2.172478735 | 2.137847269 | 2.576069243 |
|  |  |  |  |  |  |
| slackers HR | average | 63.79074367 | 63.7598296 | 62.57955709 | 65.92065391 |
|  | st.e | 3.524744145 | 4.385918123 | 5.007834444 | 3.632780314 |

*1.2 Accuracy (%)*

*LR*

| group |  | saline | 0.01mg/kg | 0.03mg/kg | 0.1mg/kg |
| --- | --- | --- | --- | --- | --- |
| all LR | average | 91.54630007 | 93.21153515 | 88.68312671 | 92.18676513 |
|  | st.e | 1.638018104 | 1.574971085 | 2.350093091 | 2.171138634 |
|  |  |  |  |  |  |
| workers LR | average | 90.416192 | 94.84977292 | 86.55712974 | 90.10238128 |
|  | st.e | 3.226041672 | 1.926785507 | 4.313080909 | 4.366799722 |
|  |  |  |  |  |  |
| slackers LR | average | 92.50254536 | 91.82533395 | 90.48204722 | 93.95047454 |
|  | st.e | 1.410421917 | 2.410417041 | 2.415999247 | 1.633440606 |

*HR*

| group |  | saline | 0.01mg/kg | 0.03mg/kg | 0.1mg/kg |
| --- | --- | --- | --- | --- | --- |
| all HR | average | 70.34107941 | 69.87697682 | 68.51959988 | 66.04094085 |
|  | st.e | 1.987795884 | 2.564226107 | 2.505821853 | 2.221633546 |
|  |  |  |  |  |  |
| workers HR | average | 71.43402742 | 71.49053478 | 72.26786992 | 68.47317433 |
|  | st.e | 2.335026098 | 3.465425331 | 3.14199147 | 3.400747521 |
|  |  |  |  |  |  |
| slackers HR | average | 69.41627725 | 68.51165854 | 65.34798677 | 63.98289714 |
|  | st.e | 3.159356388 | 3.802546017 | 3.664487741 | 2.922269998 |

*1.3 premature responses*

*LR*

| group |  | saline | 0.01mg/kg | 0.03mg/kg | 0.1mg/kg |
| --- | --- | --- | --- | --- | --- |
| all LR | average | 9.258936517 | 7.520686007 | 7.949315161 | 6.453125867 |
|  | st.e | 2.889564961 | 2.183811047 | 1.747982374 | 2.64340514 |
|  |  |  |  |  |  |
| workers LR | average | 15.09641873 | 9.211344211 | 9.373543124 | 10.53423849 |
|  | st.e | 5.834317928 | 4.11003336 | 2.952272086 | 5.219982824 |
|  |  |  |  |  |  |
| slackers LR | average | 4.319528489 | 6.090129065 | 6.744199194 | 2.999876726 |
|  | st.e | 1.051991716 | 2.142814152 | 2.098017083 | 1.828568591 |

*HR*

| group |  | saline | 0.01mg/kg | 0.03mg/kg | 0.1mg/kg |
| --- | --- | --- | --- | --- | --- |
| all HR | average | 9.717351621 | 8.461898642 | 9.472085522 | 4.267676851 |
|  | st.e | 1.63218896 | 1.719912561 | 1.514058426 | 0.735195201 |
|  |  |  |  |  |  |
| workers HR | average | 11.72120795 | 8.789197287 | 8.809708088 | 5.548217382 |
|  | st.e | 2.847378073 | 2.561746475 | 2.084213534 | 1.077894957 |
|  |  |  |  |  |  |
| slackers HR | average | 8.021780883 | 8.184953634 | 10.03255874 | 3.184142556 |
|  | st.e | 1.785221366 | 2.413656882 | 2.234126211 | 0.938008172 |

*1.4 Lever/choice latency (s)*

*LR*

| group |  | saline | 0.01mg/kg | 0.03mg/kg | 0.1mg/kg |
| --- | --- | --- | --- | --- | --- |
| all LR | average | 3.346690529 | 3.601463065 | 3.973112826 | 4.56025207 |
|  | st.e | 0.232359893 | 0.243931441 | 0.329411211 | 0.370991726 |
|  |  |  |  |  |  |
| workers LR | average | 3.494205234 | 3.587671814 | 4.295293124 | 4.992359209 |
|  | st.e | 0.401410211 | 0.343111912 | 0.588311508 | 0.66242676 |
|  |  |  |  |  |  |
| slackers LR | average | 3.221870394 | 3.613132585 | 3.700498728 | 4.194622953 |
|  | st.e | 0.273121481 | 0.35708675 | 0.355527732 | 0.391525999 |

*HR*

| group |  | saline | 0.01mg/kg | 0.03mg/kg | 0.1mg/kg |
| --- | --- | --- | --- | --- | --- |
| all HR | average | 3.190349715 | 3.384620348 | 3.504806199 | 3.756772894 |
|  | st.e | 0.17366036 | 0.198915865 | 0.169499143 | 0.197275669 |
|  |  |  |  |  |  |
| workers HR | average | 3.173813774 | 3.461854037 | 3.577732568 | 3.777738219 |
|  | st.e | 0.203660125 | 0.223604288 | 0.21396312 | 0.244439079 |
|  |  |  |  |  |  |
| slackers HR | average | 3.204341665 | 3.319268765 | 3.443099271 | 3.739033004 |
|  | st.e | 0.278262698 | 0.322453126 | 0.262081317 | 0.309062765 |

*1.5 Correct latency (s)*

*LR*

| group |  | saline | 0.01mg/kg | 0.03mg/kg | 0.1mg/kg |
| --- | --- | --- | --- | --- | --- |
| all LR | average | 0.547060792 | 0.611486595 | 0.518970963 | 0.601902779 |
|  | st.e | 0.028692862 | 0.085396167 | 0.031810284 | 0.050289902 |
|  |  |  |  |  |  |
| workers LR | average | 0.49106746 | 0.590215368 | 0.423882931 | 0.565669913 |
|  | st.e | 0.028987189 | 0.178750665 | 0.041027146 | 0.093157927 |
|  |  |  |  |  |  |
| slackers LR | average | 0.594439764 | 0.629485325 | 0.599430068 | 0.632561357 |
|  | st.e | 0.043732336 | 0.056703028 | 0.034880188 | 0.051563047 |

*HR*

| group |  | saline | 0.01mg/kg | 0.03mg/kg | 0.1mg/kg |
| --- | --- | --- | --- | --- | --- |
| all HR | average | 0.455947224 | 0.487869342 | 0.512659226 | 0.581171502 |
|  | st.e | 0.022926746 | 0.028876494 | 0.026088935 | 0.031804722 |
|  |  |  |  |  |  |
| workers HR | average | 0.406505531 | 0.444605705 | 0.469283694 | 0.514703555 |
|  | st.e | 0.02643981 | 0.047191487 | 0.028305644 | 0.032577362 |
|  |  |  |  |  |  |
| slackers HR | average | 0.497782503 | 0.524477035 | 0.549361598 | 0.63741361 |
|  | st.e | 0.032385115 | 0.033712726 | 0.039981445 | 0.047495603 |

*1.6 Collection latency (s)*

*LR*

| group |  | saline | 0.01mg/kg | 0.03mg/kg | 0.1mg/kg |
| --- | --- | --- | --- | --- | --- |
| all LR | average | 1.793409682 | 1.606538657 | 1.643455794 | 2.495213635 |
|  | st.e | 0.182180616 | 0.068473949 | 0.07036842 | 0.433382819 |
|  |  |  |  |  |  |
| workers LR | average | 1.608120635 | 1.541633117 | 1.443278988 | 2.536759019 |
|  | st.e | 0.156789934 | 0.116440632 | 0.052232707 | 0.743738107 |
|  |  |  |  |  |  |
| slackers LR | average | 1.950192722 | 1.661458729 | 1.812836168 | 2.460059848 |
|  | st.e | 0.309252753 | 0.0805102 | 0.101889009 | 0.523767519 |

*HR*

| group |  | saline | 0.01mg/kg | 0.03mg/kg | 0.1mg/kg |
| --- | --- | --- | --- | --- | --- |
| all HR | average | 1.896025008 | 1.479968861 | 1.613148239 | 1.623225838 |
|  | st.e | 0.486202078 | 0.06810326 | 0.108903797 | 0.118013898 |
|  |  |  |  |  |  |
| workers HR | average | 1.421340586 | 1.40390668 | 1.551375293 | 1.678824234 |
|  | st.e | 0.096598824 | 0.081060227 | 0.122418191 | 0.214888913 |
|  |  |  |  |  |  |
| slackers HR | average | 2.297681057 | 1.544329168 | 1.665417654 | 1.576181041 |
|  | st.e | 0.894736584 | 0.105001005 | 0.175790611 | 0.127741398 |

*1.7 Response omissions (%)*

*LR*

| group |  | saline | 0.01mg/kg | 0.03mg/kg | 0.1mg/kg |
| --- | --- | --- | --- | --- | --- |
| all LR | average | 5.166049998 | 6.471552673 | 10.73625607 | 13.14099153 |
|  | st.e | 1.32893839 | 1.55281491 | 2.015654518 | 3.44013852 |
|  |  |  |  |  |  |
| workers LR | average | 2.947658402 | 4.659090909 | 9.097569098 | 13.09917355 |
|  | st.e | 1.902582333 | 2.426194763 | 3.17544419 | 6.736333892 |
|  |  |  |  |  |  |
| slackers LR | average | 7.04315058 | 8.005174165 | 12.12283736 | 13.17637597 |
|  | st.e | 1.748176762 | 1.988401961 | 2.629942561 | 3.142998312 |

*HR*

| group |  | saline | 0.01mg/kg | 0.03mg/kg | 0.1mg/kg |
| --- | --- | --- | --- | --- | --- |
| all HR | average | 6.471215718 | 6.575557402 | 7.550615842 | 16.67019361 |
|  | st.e | 1.059740215 | 1.219860871 | 1.184817306 | 2.276332288 |
|  |  |  |  |  |  |
| workers HR | average | 5.312010986 | 4.960342611 | 5.285209252 | 13.12561736 |
|  | st.e | 1.438334055 | 1.126329339 | 1.262677113 | 2.710103894 |
|  |  |  |  |  |  |
| slackers HR | average | 7.452081261 | 7.94227761 | 9.467498342 | 19.66945043 |
|  | st.e | 1.529760575 | 2.007805832 | 1.781841976 | 3.394219203 |

*1.8 Lever/choice omissions*

| group |  | saline | 0.01mg/kg | 0.03mg/kg | 0.1mg/kg |
| --- | --- | --- | --- | --- | --- |
| all | average | 2.5 | 3.166666667 | 6.75 | 10.875 |
|  | st.e | 0.394711415 | 0.464955455 | 1.526635497 | 1.628897222 |
|  |  |  |  |  |  |
| workers | average | 1.818181818 | 3.181818182 | 5.181818182 | 10.27272727 |
|  | st.e | 0.552978412 | 0.685143407 | 1.577213779 | 2.727272727 |
|  |  |  |  |  |  |
| slackers | average | 3.076923077 | 3.153846154 | 8.076923077 | 11.38461538 |
|  | st.e | 0.52454545 | 0.658729876 | 2.48446852 | 2.024017525 |

*1.9 Completed trials*

| group |  | saline | 0.01mg/kg | 0.03mg/kg | 0.1mg/kg |
| --- | --- | --- | --- | --- | --- |
| all | average | 124.5 | 118.3333333 | 108.1666667 | 76.20833333 |
|  | st.e | 3.157691323 | 4.301022236 | 4.361253421 | 6.256874239 |
|  |  |  |  |  |  |
| workers | average | 123.1818182 | 117.3636364 | 110.5454545 | 74.18181818 |
|  | st.e | 4.534350072 | 6.320633926 | 7.276817032 | 9.48186612 |
|  |  |  |  |  |  |
| slackers | average | 125.6153846 | 119.1538462 | 106.1538462 | 77.92307692 |
|  | st.e | 4.534113519 | 6.096285285 | 5.406366325 | 8.636877952 |
